# Supplementary material for: On-Target CRISPR/Cas9 Activity Can Cause Undesigned Large Deletion in Mouse Zygotes
Source: Int J Mol Sci. 2020 May 20;21(10):3604. doi: 10.3390/ijms21103604 (PMC7279260; doi:10.3390/ijms21103604)
Supplement: Supplementary file 1 [file ijms-21-03604-s001.zip › Supplementary/Figure S1.docx]

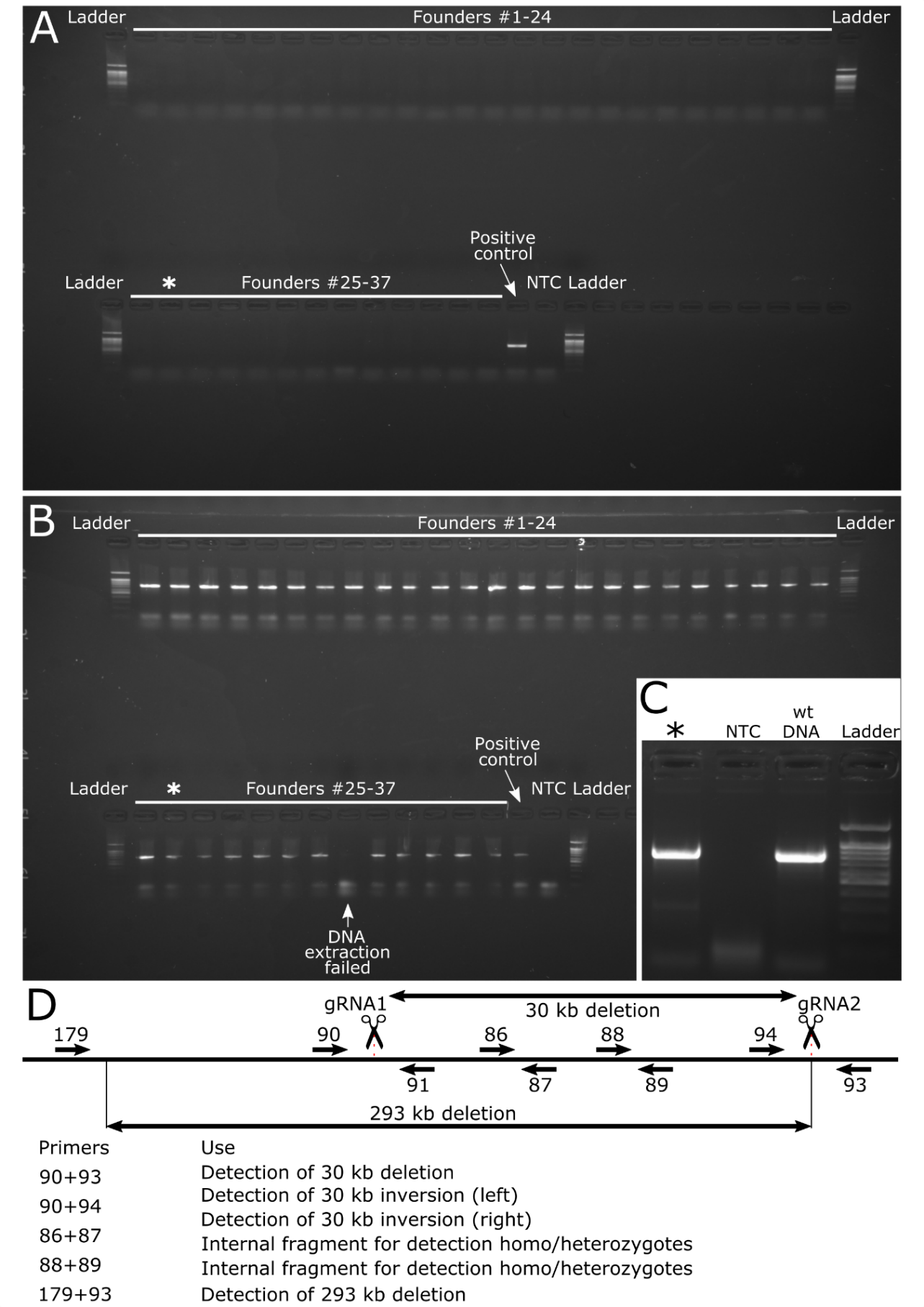


**Figure S1.** PCR detection of the rearrangements in founders. (**A**) Deletion detection: PCR with primers 90-93 detects no founders with on-target 30kb deletion. (**B**) DNA quality control: PCR with primers 88-89 detects ~700 bp band in all founders but one where DNA extraction was failed. (**C**) Integrity of the left border region: PCR with primers 90-91 shows no difference between founder with 293 kb deletion and wild type DNA sample. (**D**) Primers used for the detection of rearrangements in the analyzed locus (not to scale). *—founder with 293 kb deletion. Positive control—DNA extracted from an embryonic stem cell line with heterozygous 30kb deletion. NTC—no template control.
